# Functional trait divergence associated with heteromorphic leaves in a climbing fig

**DOI:** 10.3389/fpls.2023.1261240

**Published:** 2023-09-19

**Authors:** Jun-Yin Deng, Yong-Jin Wang, Lu-Fan Chen, Tong Luo, Rong Wang, Xiao-Yong Chen

**Affiliations:** ^1^Zhejiang Tiantong Forest Ecosystem National Observation and Research Station, School of Ecological and Environmental Sciences, East China Normal University, Shanghai, China; ^2^Shanghai Institute of Pollution Control & Ecological Security, Shanghai, China

**Keywords:** ecophysiology, functional differentiation, heteroblasty, leaf economics spectrum, photosynthetic characteristics

## Abstract

**Introduction:**

Plants that display heteroblasty possess conspicuous variations in leaf morphology between their juvenile and adult phases, with certain species retaining juvenile-like leaves even in adulthood. Nevertheless, the ecological advantages of maintaining two or more distinct leaf types in heteroblastic plants at the adult stage remain unclear.

**Method:**

The aim of this study is to examine the adaptive significance of heteroblastic leaves sampled from branches with divergent functions (sterile and fertile branches) of mature *Ficus pumila* individuals by comparing their morphological, anatomical, and physiological characteristics.

**Result:**

Leaves on sterile branches (LSs) exhibited a significantly larger specific leaf area, thinner palisade and spongy tissues, lower chlorophyll contents, and lower light saturation points than leaves on fertile branches (LFs). These results demonstrate that LSs are better adapted to low light environments, while LFs are well equipped to take advantages of high light conditions. However, both LFs and LSs have a low light compensation point with no significant difference between them, indicating that they start to accumulate photosynthetic products under similar light conditions. Interestingly, significant higher net photosynthetic rate was detected in LFs, showing they have higher photosynthetic capacity. Furthermore, LFs produced significant more nutrients compared to LSs, which may associate to their ability of accumulating more photosynthetic products under full light conditions and higher photosynthetic capacity.

**Discussion:**

Overall, we observed a pattern of divergence in morphological features of leaves on two functional branches. Anatomical and physiological features indicate that LFs have an advantage in varied light conditions, providing amounts of photosynthetic products to support the sexual reproduction, while LSs adapt to low light environments. Our findings provide evidence that heteroblasty facilitates *F. pumila* to utilize varying light environments, likely associated with its growth form as a climbing plant. This strategy allows the plant to allocate resources more effectively and optimize its overall fitness.

## 1 Introduction

As a photosynthetic organ, leaves are crucial for plants, and their diversity in the appearance indicates the disparity in the ability to perform photosynthesis, which can ultimately impact plants’ fitness (Dani et al., 2020; Momayyezi et al., 2022; Sun et al., 2023). Mature leaves usually display consistent traits, and their economic spectrum reflects the optimal allocation of resources based on the functional requirements of plants (Wright et al., 2004; Bartholomew et al., 2022; Joswig et al., 2022; Sun et al., 2023), affecting the survival of plants in different environments (Kumordzi et al., 2019; Lopez et al., 2021; Wang et al., 2022). Therefore, it is crucial to uncover the ecological importance of leaf morphological differences to understand how well plants can adapt to changes in their surroundings.

In some plants, leaf traits (particularly the shape and size) can change in response to environmental shifts (e.g., shift from aquatic to terrestrial environments), known as heterophylly (Nakayama et al., 2017). For example, leaves of *Hygrophila difformis* in submerged aquatic environments are deeply dissected, while they are simply marginally serrated in terrestrial environments (Li et al., 2017). Besides heterophylly, leaf shape or/and size of certain plants undergo significant changes during their developmental stages (Zotz et al., 2011), commonly called heteroblasty. Such as leaves of juvenile *Pinus cembroides* are concave, flattened and needle-shaped, while leaves of adults are triangular or semicircular needle-shaped (Webster et al., 2022). The significant alteration of leaves during various stages of individual growth suggests that plants are optimizing their resource allocation to adapt to drastic environmental changes during their development (Westoby et al., 2022). However, this phenomenon has received little attention.

Heteroblasty had been reported and described in many groups including trees, bromeliads, herbs and climbing plants (Zotz et al., 2011). Heteroblasty usually evolves as a result of adaptation to predictable environmental changes, particularly changes in the gradient of light (Rose et al., 2019), which is especially true for some climbing plants (Beyschlag and Zotz, 2017). Juveniles of climbing plants often grow under the canopy characterized by low light intensity. As they become mature, they eventually reach the canopy where light intensity is high (Zotz et al., 2011). As a result, leaf traits of these plants transit from skototropism to phototropism as they move from their juvenile to adult stages (Brito et al., 2022). Recently, Ehmig et al. (2019) revealed heteroblastic restios represented a response to temporal environmental shifts from moist conditions to harsher ones. Practically, their findings showed that certain neotenous species retained vegetative shoots, which indicates a preference for wetter conditions and fertile soils compared to non-neotenous species (Ehmig et al., 2019). Nevertheless, it is still uncertain what the morphological, anatomical, and physiological differences are between the leaves of sterile and reproductive shoots, particularly in adult neotenous species.

*Ficus pumila*, a climbing fig species, is a woody evergreen vine commonly found in a subtropical southeastern China (Chen et al., 2012). It has two varieties, *F. pumila* var. *pumila* and *F. pumila* var. *awkeotsang*. The former is extensively distributed across mainland Asia and the island of Taiwan, whereas the latter is primarily found in Taiwan, with only a few occurrences in Fujian and Zhejiang provinces (Wu & Raven, 1994). Both varieties only have sterile branches before the reproductive stage (Ferrer-Gallego et al., 2015), and the fertile branches will grow once they reach to reproductive stage. Typically, fertile branches extend outward and are positioned above the sterile branches that are closely attached to the plant or other objects using an adventitious root (Ferrer-Gallego et al., 2015). In addition, the leaves on the fertile branches are generally larger than those on the sterile branches. The dissimilarities in the morphological characteristics of leaves observed between different species or individuals, usually indicate variation in their physiological functions, particularly their capability to perform photosynthesis (Luo et al., 2021; Momayyezi et al., 2022; Sun et al., 2023). However, it remains unclear whether two morphological types of leaves on the same individual possess dissimilar photosynthetic abilities.

The sterile branches of *F. pumila* are located lower than the fertile branches, suggesting that the LSs grow in areas with low light intensity. As a result, we propose that LSs may exhibit characteristics similar to those of shade leaves, including a greater specific leaf area, a lower light compensation point, and a lower net photosynthetic rate (Mathur et al., 2018). Furthermore, figs or syconia only develop on fertile branches, which requires a significant amount of resources, primarily generated through photosynthesis, to support their growth. Since photosynthetic substances are primarily produced by leaves and transported over short distances (Castorena et al., 2022), it is likely that the LFs are responsible for supplying the resources required for the growth of figs (Li et al., 2019). Therefore, it is reasonable to predict photosynthesis capacity of LFs is higher than that of LSs. In order to test those hypotheses, we quantified and compared the morphological, anatomic, and physiological differences between LF and LS. Particularly we ask: 1) Do LFs and LSs significantly differ in their morphology, anatomy and physiology? 2) Whether LFs have significant higher photosynthetic capacity than LSs?

## 2 Materials and methods

### 2.1 Study sites and plant materials

The study sites were located in Tiantong National Forest Park (29.73-29.83°N, 121.69-121.83°E) and adjacent areas of Zhejiang, China. The dominant vegetation of the study area is evergreen board-leaved forests. All the measurements in fields and experiments in the lab were performed in July-August 2020.

**Figure 1:**
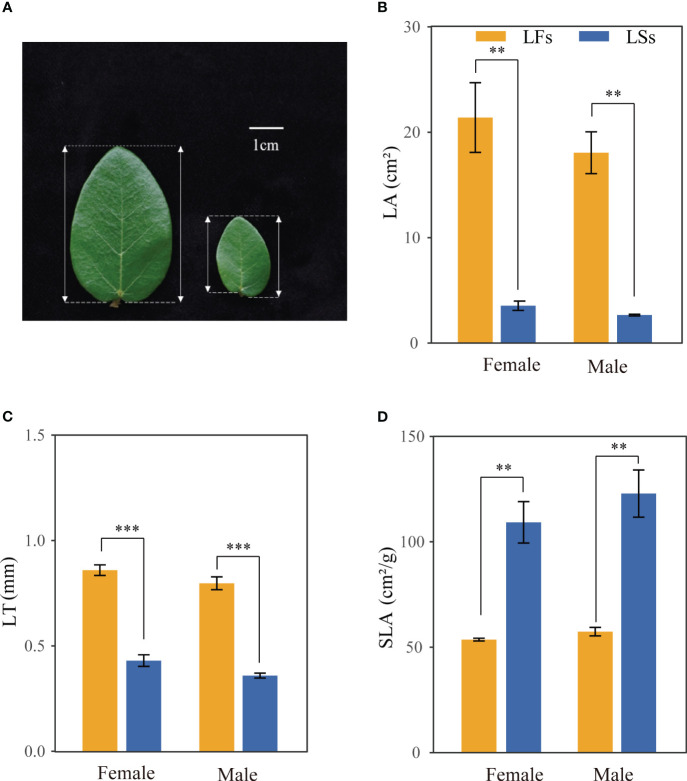
Morphological features of leaves on fertile (LFs) and sterile (LSs) branches. **(A)** leaf on fertile branch (left) and sterile branch (right) **(B)** Leaf area; **(C)** Leaf thickness; **(D)** Specific leaf area. **: *P*<0.01, ***: *P*<0.001; LFs, leaves on fertile branches; LSs, leaves on sterile branches.

We chose *F. pumila* var. *pumila* to conduct experiments due to its extensive geographic range and accessibility. *Ficus pumila* var. *pumila* (hereafter *F. pumila*), an evergreen broad-leaved climber, is usually found on the wall or tree. *F. pumila* relies on its specific pollinators (*Wiebesia pumilae*) to pollinate (Chen et al., 2012; Wang et al., 2021). The juvenile individual has only sterile branches, relying on the adventitious root to climb and reach high. Once the individual matured, it produces fertile branches, where the syconia or figs are born. The fertile and sterile branches bear leaves distinct in morphology (**Figure 1A**). Mature leaves on sterile branches are ovate-cordate with a short petiole, and asymmetrical leaf base, while mature leaves on fertile branches are ovate-elliptic with a long petiole and roughly symmetrical base (Ferrer-Gallego et al., 2015) (**Figure 1A**).

*F. pumila* is a functionally dioecious fig species. To reveal trait consistency between male and female individuals, we randomly selected five individuals for each gender in the field to perform the experiments. All the measurements and lab work were performed on both genders.

### 2.2 Measurement of leaf morphological traits

We randomly sampled five mature leaves on fertile and sterile branches per tree. For each leaf, we weighed in fresh for the fresh weight (FW, in g), and measured leaf thickness (LT, in mm) using a Syntek thickness gauge (Deqing Shengtaixin Electronic Technology Co., Ltd, China). In order to measure leaf area (LA, in cm^2^), we first scanned each leaf using the scanner (HP, M127128, HP, USA) and then measured the scanned image using imageJ v3.1.2 (Abràmoff et al., 2004). After that, we dried the leaves for 48 hours for the dry weight (DW, in g). The specific leaf area (SLA, in cm^2^·g^-1^) was calculated based on LA and DW.

### 2.3 Anatomical measurement

In order to reveal the micro-structure of two leaf types, we randomly sampled 30 mature leaves for each type (three leaves for each leaf type per tree on both male and female individuals) with a total of 60 mature leaves. Sampled leaves were immediately put into 50 ml tubes (with leaves of the same type sampled from one tree stored separately) with 70% FAA solution (the ratio of formalin, acetic acid, and 70% ethanol is 1:1:18) and kept for more than 24 hours. Following that, the whole leaf was taken out from 70% FAA solution, and then dehydrated by sequential one-hour incubations in 75%, 85%, 95%, and 100% ethanol. After that, the dehydrated leaves were decolorized with a solution that includes absolute ethanol and dimethylbenzene with a ratio of 1:1 for 1 hour. The decolorized leaves were set and embedded in wax. The embedded sample was then sliced manually with the Leica RM2235 microtome (Leica, Germany) into 2-4 μm sections. Well-sliced sections for each leaf were stained with hematoxylin-eosin for one minute and photographed using Leica DIM800 (Leica, Germany). For each photograph, we randomly selected three visual fields, measured the thickness of different tissues, and counted the number of cell layers for each tissue using the Digmizer version 6.0.0 (MedCalc Software, China). Parameters of different tissues in each photographed section were represented by the average value of the three visual fields.

### 2.4 Measurement of leaf physiological properties

We used a portable gas-exchange analyzer LI-6800 (Li-CoR, Lincoln, Nebraska, USA) to measure photosynthetic parameters. The measurement was performed for two leaf types on selected individuals with three repeats for each leaf type per tree. The measurement was conducted from 8:30 to 11:30 am in sunny days in July 2020 with a standard environmental condition (temperature, 25°C; CO_2_ concentration, 400 μmol·mol^-1^) of the leaf chamber. Different light intensity was set for the two leaf types because of the large difference in light saturation points based on the pilot experiments. Specifically, the light intensity was in sequence of 1200, 1000, 900, 800, 700, 600, 500, 400, 300, 200, 100, 50, and 0 μmol photons m^-2^·s^-1^ for LS, while it was changed in the sequence of 2400, 2200, 2000, 1800, 1500, 1200, 800, 600, 400, 200, 100, 50, 25, 0 μmol photons m^-2^·s^-1^ for LF. The steady-state rates at each light level were recorded. After that, we used four light response models (rectangular hyperbola (Baly, 1935), non-rectangular hyperbolic (Thornley, 1976), exponential (Bassman and Zwier, 1991), and modified rectangular hyperbolic model (Ye, 2007; Ye et al., 2012)) to fit the light response curve to understand the photosynthetic features. We calculated R^2^ for each model to compare and select the best one (with the largest R^2^). The exponential model (equation 1) fitted best, and was used to estimate photosynthetic parameters including light compensation point (LCP), maximum net photosynthetic rate (Pn_max_), light saturation point (LSP, about 0.99 Pnmax according to Wang et al. (1991)) and dark respiration rate (Rd).


(Equation 1) $$\mathrm{Pn}=\text{P}n_{\text{max}}\left(1-{\text{e}}^{-\text{b}\left(\mathrm{PAR}-\mathrm{LCP}\right)}\right)$$

where *Pn* is the net photosynthetic rate, *PAR* is the photosynthetically active radiation.

Following that, we extracted transpiration rate (Tr), intercellular CO_2_ concentration (Ci), and stomatal conductance (Gs) under LSP and calculated water use efficiency (WUE, Pn/Tr) for each leaf type. The exponential model was used to fit the light response photosynthetic curve and the photosynthetic parameters were estimated based on this model, which was performed in R v4.0.3 using minpack.lm packages (Elzhov et al., 2010).

Chlorophyll contents (chlorophyll a, Chl-a; chlorophyll b, Chl-b; total chlorophyll content, Chl-(a+b)) were measured following the standard protocols of Mackinney (1941) using the acetone method. To simplify, the leaf sample was cut after removing the main vein, and 0.1 g samples were weighted and grounded using a mortar. Then, the well-grounded sample was transferred into a 50 mL glass tube, adding 80% acetone to 30 ml. Tubes with grounded samples and 80% acetone were wrapped with tin foil and set overnight in a dark place. Finally, we measured the absorbances of the solution under 663 (A663) and 645 nm (A645), respectively, using a SpectraMax Multi-Mode Microplate Reader M4 (MolecularDevices, USA). The concentrations of chlorophyll components were calculated based on the absorbances, acetone volume, and sample weight.

Soluble sugars were quantified by the anthrone method (Yemm and Willis, 1954). Simply, both tested and blank solutions were prepared first, then the absorbance of the prepared solution under 620 mm was measured using a SpectraMax Multi-Mode Microplate Reader M4 (MolecularDevices, USA). The soluble sugar content was calculated according to the following equation.


(Equation 2) $$\text{Soluble sugar content }(\text{mg}/\text{g})\;=\;\frac{2.34\times \left(△\text{A}+0.07\right)}{\text{W}}$$

where *W* is the fresh weight of the sample, *ΔA* is the difference between the tested and the blank solution. Soluble sugar content per unit mass was converted into the content per unit area.

The content of soluble proteins was quantified by the bicinchoninic acid method (BCA, Smith et al., 1985) using a protein measurement kit (Suzhou Keming Biological Co., Ltd.) following the standard protocols. Tested, blank and standard solutions were first prepared, then the absorbance of the prepared solution under 562 mm was measured using a SpectraMax Multi-Mode Microplate Reader M4 (MolecularDevices, USA). The protein content was calculated based on the absorbed values of those solutions by equation 3.


(Equation 3) $$\text{Protein content }\left(\text{mg/g}\right)=\frac{{\text{W}}_{\text{s}}\;\times \;\left({\text{A}}_{t}\;-\;{\text{A}}_{\text{b}}\right)}{{\text{W}}_{\text{t}}\;\times \;\left({\text{A}}_{\text{s}}\;-\;{\text{A}}_{\text{b}}\right)\;}$$

where *W_s_* and *W_t_* were the weight of standard protein and tested samples, *A_t_*, *A_b_* and *A_s_* were absorbed values of tested, blank and standard solution. The content of soluble proteins per unit mass was converted into the content per unit area.

### 2.5 Statistical analysis

To test the differences in the parameters between LF and LT or between male and female individuals, we used the t-test. If the data did not conform to a normal distribution, a non-parameter test (Wilcoxon rank sum test) was carried out. In order to examine the relationship between parameters of leaf morphological, anatomic, and physiological traits, the Spearman test method was used for correlation analysis. All statistical analysis and graphing were performed with the R v 4.0.3 (R Core Team, 2022).

## 3 Results

### 3.1 Morphological and anatomic differences of two leaf types

Significant differences are observed in morphology between the two leaf types of *F. pumila*. Specifically, leaves on fertile branches (LFs) have a significantly larger leaf area (LA) than those on sterile branches (LSs), but a smaller specific leaf area (SLA) of both male and female individuals (**Figures 1B, D**). LFs (female: 0.859 ± 0.050 mm, male: 0.788 ± 0.057 mm) are significantly thicker than that of LSs (female: 0.430 ± 0.055 mm, male: 0.299 ± 0.114 mm) (**Figure 1C**). No significant difference was found between LSs and LFs on male and female trees, respectively.

**Figure 2:**
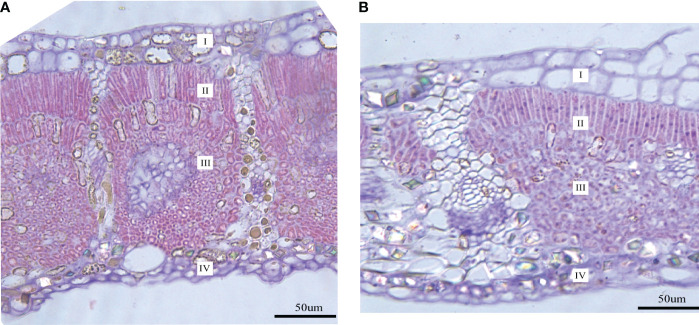
Transverse section images of mature leaves on fertile and sterile branches. **(A)** The leaf transverse section on the fertile branch; **(B)** The leaf transverse section on the sterile branch. I, upper epidermis; II, palisade tissue; III, spongy tissue; IV, lower epidermis.

Both LFs and LSs have four tissue types (upper and lower epidermis, palisade and spongy tissues) (**Figure 2**). Significant differences are found in palisade and spongy tissues, while no significant difference is detected in the upper and lower epidermis between LFs and LSs (**Table 1**). Specifically, palisade and spongy tissue in LFs are significantly thicker than in LSs, which may because that both tissues in LFs are composed of more layers of cells (**Table 1**). The anatomic charterers between LSs and LFs on male and female trees are consistent, respectively (**Table 1**).

**Table 1:** Comparison of anatomical structure characteristics between leaves on fertile (LFs) and sterile branches (LSs).

		Female		Male	
		LFs	LSs	LFs	LSs
Upper epidermis	Thickness (μm)	44.89 ± 4.47^a^	41.00 ± 1.50^a^	44.61 ± 5.34^a^	38.33 ± 2.94^a^
	layers	2-3	2	2-3	2
Palisade tissue	Thickness (μm)	66.04 ± 8.23^a^	39.25 ± 5.19^b^	49.21 ± 4.13^a^	32.99 ± 1.27^b^
	layers	2	1	2	1
Spongy tissue	Thickness (μm)	156.63 ± 31.37^a^	96.01 ± 17.38^b^	162.13 ± 29.88^a^	80.15 ± 13.97^b^
	layers	16-19	11-13	17-20	10-14
Lower epidermis	Thickness (μm)	33.91 ± 5.02^a^	32.65 ± 7.16^a^	36.53 ± 7.36^a^	31.06 ± 9.29^a^
	layers	3-4	2-3	3-4	2-3

The values of different thickness are mean ± standard deviation (SD), and different alphabet of top right-hand corner in the same column indicates a significant difference of P<0.05.

### 3.2 Physiological differences between two leaf types

**Figure 3:**
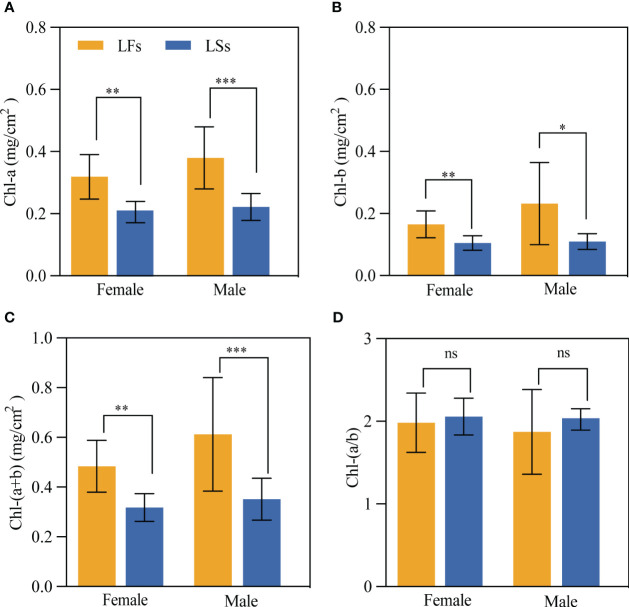
The comparison of Chlorophyll content of leaves on fertile and sterile branches. **(A)** Chlorophyll a; **(B)** Chlorophyll b; **(C)** Total chlorophyll; **(D)** Chlorophyll a/b ratio. *: *P*<0.05, **: *P*<0.01, ***: *P*<0.001; LFs, leaves on fertile branches; LSs, leaves on sterile branches.

All chlorophyll contents (Chl-a, Chl-b, and Chl-(a+b) of LFs are significantly higher than that in LSs (**Figure 3**). And the average chlorophyll contents in LFs are around 1.5 and 2 times higher in male and female trees respectively, compared to LSs. We find no significant difference in all chlorophyll contents between male and female trees. Interestingly, similar level of chlorophyll a/b ratio was detected between LFs and LSs (**Figure 3D**), showing that both LFs and LSs have similar ability of using low light.

**Figure 4:**
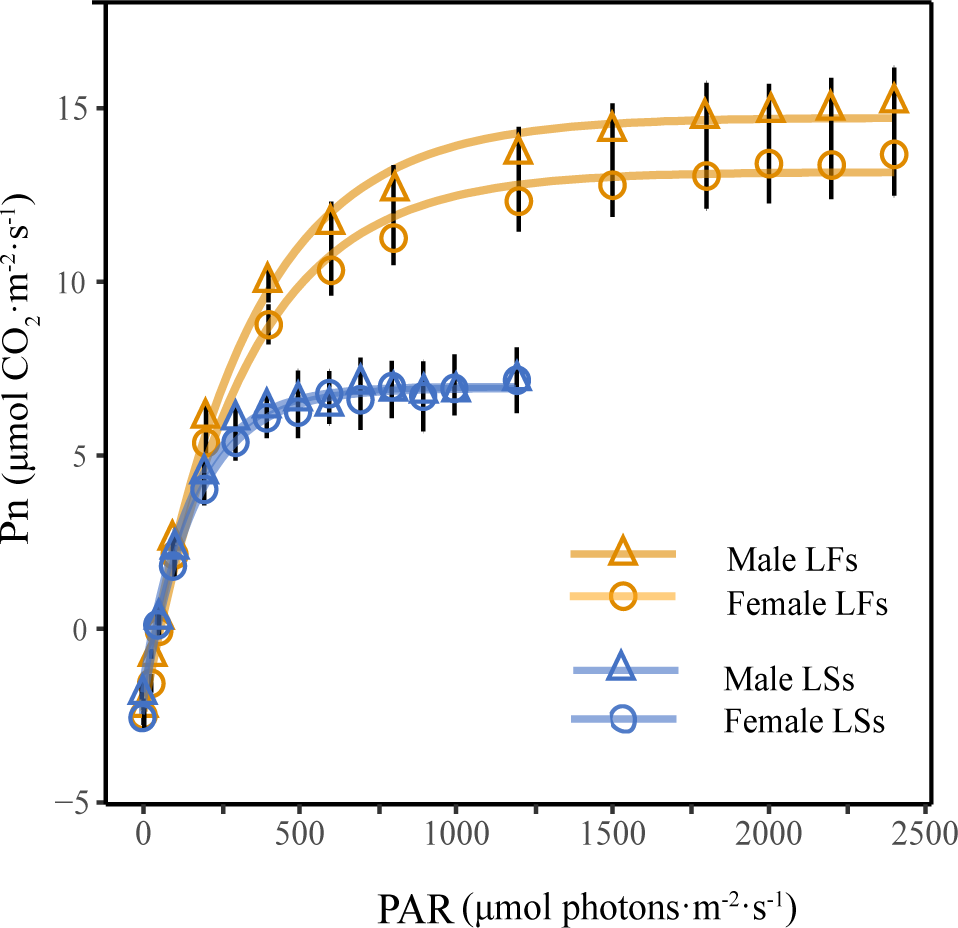
Light response curves of leaves on fertile and sterile branches. LFs, leaves on fertile branches; LSs, leaves on sterile branches.

The exponential model is the best model for simulating photosynthetic light response curves of two leaf types, and its determining coefficients (R^2^) are larger than 0.99 (*P*< 0.001). The light response curves of LFs and LSs show that the net photosynthetic rates (Pn) of both leaves increased along with light intensity (**Figure 4**). There is no obvious difference in Pn between LFs and LSs when PAR (photosynthetically active radiation)< 200 μmol·m^-2^·s^-1^. As PAR increases, the differences in Pn increase between the two leaf types. The Pn of LSs tends to stabilize when PAR reached 500 μmol·m^-2^·s^-1^, while Pn of LFs tends to stabilize around 1500 μmol·m^-2^·s^-1^. For each leaf type, there is no significant difference in the light response curves between female and male trees.

Significantly higher LSP and Pn was detected in LFs than in LSs of both male and female individuals, revealing LFs have an advantage under high light conditions (**Table 2**). Surprisingly, the light compensation point (LCP) and dark respiration rate (Rd) show no significant difference between the two types of leaves (**Table 2**), suggesting that both LFs and LSs start to accumulate photosynthetic products under same light conditions. The photosynthetic parameters of each leaf types are similar between male and female trees.

**Table 2:** Photosynthetic parameters of leaves on fertile (LFs) and sterile branches (LSs).

	Female		Male	
Parameters	LFs	LSs	LFs	LSs
LCP (μmol photons·m^-2^·s^-1^)	51.34 ± 21.46^a^	49.50 ± 26.01^a^	41.60 ± 12.81^a^	43.92 ± 8.15^a^
LSP (μmol photons·m^-2^·s^-1^)	1548.73 ± 105.78^a^	919.12 ± 368.98^b^	1553.26 ± 204.04^a^	806.36 ± 240.73^b^
Pnmax (μmol CO_2_·m^-2^·s^-1^)	13.15 ± 2.82^a^	7.03 ± 3.10^b^	14.67 ± 2.57^a^	7.04 ± 2.09^b^
Rd (μmol CO_2_·m^-2^·s^-1^)	2.42 ± 0.43^a^	2.45 ± 1.09^a^	2.06 ± 0.53^a^	1.89 ± 0.57^a^

The values of different thickness are mean ± standard deviation (SD), and different alphabet of top right-hand corner in the same column represented significant differences, *P*<0.05. LCP, light compensation point; LSP, light saturation point; Pnmax, maximum net photosynthetic rate; Rd, dark respiration rate.

Significant higher Tr was detected in LFs (female: 7.706 ± 3.876 mmol·m^-2^·s^-1^, male: 7.898 ± 3.450 mmol·m^-2^·s^-1^) compared to LSs (female: 3.784 ± 1.303 mmol·m^-2^·s^-1^, male: 2.796 ± 1.172 mmol·m^-2^·s^-1^) (**Table 3**). Gs of LFs is about two and three times larger than those of LSs of females and males, respectively (**Table 3**). Those results illustrate that LFs may have stronger transpiration pull, thus facilitating the transportation of water and nutrients to fertile branches. No significant difference in water use efficiency (WUE) and intercellular CO_2_ concentrations (Ci) was found between LFs and LSs (**Table 3**). And there is no significant difference either in gas exchange parameters between female and male trees for each type of leaf.

**Table 3:** Instantaneous gas exchange parameters near the light saturation point of leaves on fertile (LFs) and sterile branches (LSs).

	Female		Male	
Parameters	LFs	LSs	LFs	LSs
Tr (mmol·m^-2^·s^-1^)	7.71 ± 3.88^a^	3.78 ± 1.30^b^	7.90 ± 3.45^a^	2.80 ± 1.17^b^
Gs mol·m^-2^·s^-1^)	0.24 ± 0.13^a^	0.10 ± 0.06^b^	0.25 ± 0.07^a^	0.08 ± 0.04^b^
WUE (μmol·mmol^-1^)	2.05 ± 0.20^a^	1.84 ± 0.53^a^	2.11 ± 0.76^a^	1.56 ± 0.38^a^
Ci (μmol·mol^-1^)	257.29 ± 15.51^a^	276.05 ± 29.02^a^	253.25 ± 16.97^a^	284.61 ± 38.19^a^

The values of different thickness are mean ± standard deviation (SD), and different alphabet of top right-hand corner in the same column represented significant differences, *P*<0.05. Tr, transpiration rate; Gs, stomatal conductance; WUE, water use efficiency; Ci, intercellular CO_2_ concentration.

**Figure 5:**
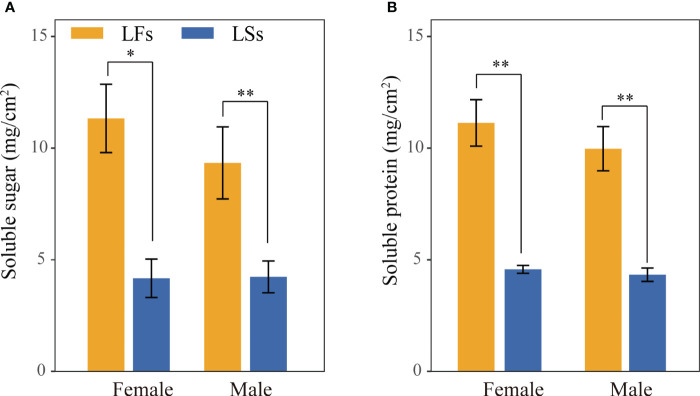
Nutrient content comparison of leaves on fertile and sterile branches. **(A)** soluble sugar; **(B)** soluble protein. *: *P*<0.05, **: *P*<0.01; LF, leaves on fertile branches; LS, leaves on sterile branches.

In general, LFs produce significantly more nutrients compared to LSs of both male and female individuals, which may associate to their high photosynthetic capacity (**Figure 5**). The average soluble sugar and protein content in LFs is over two times higher than LSs of females and males, respectively (**Figure 5**). Similar levels of nutrients were observed in each leaf type of male and female trees.

### 3.3 Relationships between morphological, anatomic, and physiological characteristics

**Figure 6:**
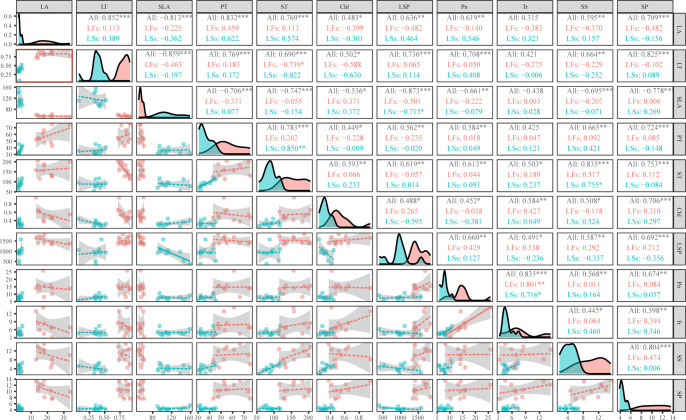
Correlation analysis of phenotype, anatomical structure, and physiological characteristics of leaves on fertile and sterile branches. The diagonal part shows the distribution diagram, the lower left part shows the bivariate scatter diagram with fitting lines, and the upper right part shows the correlation coefficient and significance level. LA, leaf area; LT, leaf thickness; SLA, specific leaf area; PT, palisade parenchyma; ST, spongy mesophyll; Chl, total chlorophyll content; LSP, light saturation point; Pn, maximum net photosynthetic rate; Tr, transpiration rate; SS, soluble sugar; SP, soluble protein; *: *P*<0.05, **: *P*<0.01, ***: *P*<0.001; LFs, leaves on fertile branches; LSs, leaves on sterile branches, All, all leaves.

Interestingly, morphological parameters are divergent between LFs and LSs, while the range of all anatomic and physiological parameters (except for LSP) of LFs cover that of LSs, implying that LFs can use wider ranges of resources. And there is almost no significant relationship between most parameter pairs (54 and 51 out of 55 pairs for LFs and LSs, respectively) within each leaf type (**Figure 6**). When combining LFs and LSs, leaf phenotype (leaf area and thickness), anatomical structure (thickness of palisade and sponge cells), and physiological characteristics parameters (total chlorophyll content, photosynthetic parameters, and nutrients) are mostly positively correlated except for SLA, which is negatively correlated to the rest of parameters (**Figure 6**). Tr is only positively related to physiological parameters and ST.

## 4 Discussion

Leaves on sterile and fertile branches of mature *F. pumila* individuals have significant differences in terms of morphology, anatomy, and physiology. LFs and LSs are genetically identical, because they were sampled from same individual. Therefore, such differences are likely to be an adaptation to distinct environmental conditions, particularly the long-term exposure to varying light intensities experienced by leaves of sterile and fertile branches of *F. pumila*. Generally, LSs are better equipped to capture light in low light conditions as they have a significantly larger specific leaf area, small chlorophyll a/b ratio and low light compensation point. Contrary to LSs, LFs are well adapted to high-light environments as they have smaller specific leaf area, large chlorophyll contents and high light saturation points. The higher photosynthetic ability of LFs allows them to produce more organic matters, implying that LFs may have invested more resources in reproduction, which may be the consequence of functional divergence between the two branches.

As a typical climbing plant, *F. pumila* experiences shifts in light regimes during the development despite the fact that it can thrive in a variety of environments, such as subtropical forests, open lands with rocks, or abandoned villages (Liu et al., 2013). LSs are produced on both juvenile and mature *F. pumila* individuals, which are usually sheltered by other plant’s leaves or LFs (personal observations). As a result, LSs are less likely to be exposed to high light intensity, implying that they have been selected to adapt to low light conditions. This is supported by evidence from morphological, anatomic and physiological characters of LSs.

Leaves under low light environments generally have large leaf areas (Valladares et al., 2016; Ntawuhiganayo et al., 2020), though there are some exceptions (Delagrange et al., 2006; Sebuliba et al., 2022). A large leaf means large area to capture light in situations where light intensity is low. This can also be accomplished by decreasing the number of overlapping leaves through the reduction of leaf angles on branches (Valladares and Niinemets, 2008; de Haldat du Lys et al., 2023). Though their areas in *F. pumila* are quite small, LSs are located on sterile branches that are oriented horizontally, with minimal overlapping (personal observations). Besides, small leaves are of low cost compared to maintaining a larger and more complex photosynthetic organ. Furthermore, the SLA of LSs in *F.pumila* is notably greater than that of LFs. In conditions of low light intensity, plants tend to dedicate more resources towards enlarging the surface area of their leaves and enhancing their capacity to capture light. Therefore, SLA of plants at low light conditions is generally high (Sevillano et al., 2016; Pitchers et al., 2021; Tang et al., 2022). For example, Paź-Dyderska et al. (2020) measured the SLA of the sunny and shaded leaves of 179 woody plants, except for a very few species, the SLA of the shade leaves in most plants is greater than that of full-light leaves.

The adaptation of leaf to changes in light environments leads to changes in its anatomical characteristics. Particularly, as light intensity decreases, leaf thickness, thickness of palisade and spongy tissues decrease, and the number of palisade tissue layers decreases (Putz and Mooney, 1991), which are adaptations of plants to low light environments. For example, Nascimento et al. (2015) revealed that leaf and mesophyll thickness was greater under high light intensity in *Eugenia hiemalis*. LSs is significantly thinner than LFs, and it also has fewer palisade and spongy tissues, suggesting it adapts to low light environments.

Regarding physiological traits, the Chl a/b ratio is one of important indicator for shade tolerance of plant species. The Chl a/b ratio of LSs in *F. pumila* is about 2, which is like most shade-tolerant plant species where the ratio is usually less than 3 (Lichtenthaler et al., 1981; Hoflacher and Bauer, 1982). Furthermore, plants that are adapted to low-light environments usually have lower light compensation point (Sterck et al., 2013; Sakanishi et al., 2022; Zhang et al., 2022). This means that the plants begin to show net accumulation in organic matters at relatively low light intensities. Therefore, they can use low light quantum density to their maximum capacity under limited light conditions, maximizing photosynthesis and increasing organic matter accumulation to meet their energy needs for survival and growth. The LCP for shade leaves can range from as low as 5-20 µmol photons m^-2^·s^-1^ to around 50-100 µmol photons m^-2^·s^-1^ (DeLucia et al., 1996; Karabourniotis et al., 2021). For example, the LCP of *Acer davidii* (shade tolerant species) is about 80 µmol photons m^-2^·s^-1^ under full sunlight (Lin and Liu, 2008), while shade tolerant *Hemiboea rubribracteata* is about 9.34 µmol photons m^-2^·s^-1^ (Li et al., 2015). LSs of *F. pumila* have an LCP of 50 µmol photons m^-2^·s^-1^, which is within the range for shade tolerant species, indicating that they can utilize low light intensity efficiently.

The dissimilarities in leaf morphology between LFs and LSs are associated with their physiological variation, particularly in terms of the photosynthetic capacity (Etnier et al., 2017). Within species, the rate of photosynthesis is positively correlated with the leaf mass per area (LMA, 1/SLA), especially the photosynthetic mass (Osnas et al., 2018). The palisade and spongy tissues containing chloroplast cells are major contributors to the photosynthetic mass of leaves (Fan et al., 2019). Han et al. (2017) and Xie et al. (2015) have also shown that a higher number of chloroplasts corresponds to a greater photosynthetic capacity. In line with that, our study found that Pn is significantly higher in leaves with more palisade and spongy tissue (LFs) compared to those with less (LSs). Additionally, The LSP is notably higher in LFs than LSs, which could be attributed to the higher level of chlorophyll in LFs, which contains more palisade and spongy tissue compared to LSs. This higher photosynthetic capacity is linked to the more organic substrates found in LFs, congruent with the requirement of high levels of nutrients for reproduction. Thus, the difference in photosynthetic capacity between LSs and LFs in *F. pumila* may be due to functional divergence of sterile and fertile branches.

During the vegetative stage, *F.pumila* utilizes sterile branches to reach the forest canopy or expand its territory in open areas. This process requires rapid growth, which suggests that the leaves on sterile branches employ acquisitive strategies with fast investment and return (Medina-Vega et al., 2021; Niklas et al., 2023). Fertile branches are responsible for producing figs to exert the sexual reproduction of *F. pumila* or to support the development of its specific pollinators (Liu et al., 2014). In addition, male and female fig plants contain hundreds to thousands of fig wasps and seeds, respectively, which need a lot of nutrients to grow (Aguirre et al., 2018). As a result, leaves on fertile branches that can produce more photosynthetic products may be preferred by natural selection. In supporting that, we found LFs are large and thick with high LSP and Pn (**Table 2**). More importantly, LFs have similar LCPs and Chl a/b ratio with LSs (**Figure 3D**), implying that LFs can also accumulate photosynthetic products in low light conditions. These characteristics allow LFs to produce a significantly more amount of carbohydrates through photosynthesis at a wide range of light conditions. These carbohydrates are then transported to nearby figs to support sexual reproduction or to aid the development of pollinating fig wasps. In addition, higher Tr and Gs observed in LFs (**Table 3**) suggest that they can provide a powerful transpiration pull, which helps the leaves obtain an adequate supply of water and inorganic salts. This, in turn, enhances the LFs’ photosynthetic ability and allows them to accumulate more photosynthetic products that are essential for sustaining reproduction of *F. pumila*.

*Ficus pumila* is typically found in subtropical forests (Bain et al., 2015), where the understory has low light intensities. As a result, it is difficult for *F. pumila* to produce sufficient organic matters for sexual reproduction or development of pollinating fig wasps. However, *F. pumila* can maximize its organic product production by utilizing the full light available above the forest canopy with the leaves on its fertile branches. This allows *F. pumila* to meet the nutrient requirements for sexual reproduction and nursing fig wasps, thereby sustaining their obligate mutualistic relationship. By functionally differentiating between sterile and fertile branches, *F. pumila* enhances its fitness in utilizing the varying light intensities in microhabitats and optimizing its resource allocation. In addition, with rapid growth of sterile branches, *F. pumila* can extend its fertile branches above the canopy to place figs in better conditions, which can facilitate pollination by increasing pollinator dispersal distance or seed dispersal by attracting large frugivores with greater dispersal abilities.

## Data availability statement

The dataset has been made publicly available through Dryad and can be accessed using the following DOI: 10.5061/dryad.d7wm37q6j.

## Author contributions

J-YD: Conceptualization, Writing – original draft. Y-JW: Data curation, Formal Analysis, Methodology, Software, Visualization, Writing – review & editing. L-FC: Visualization, Writing – original draft. TL: Methodology, Visualization, Writing – review & editing. RW: Validation, Writing – review & editing. X-YC: Conceptualization, Funding acquisition, Supervision, Validation, Writing – review & editing.
